# Efficacy and safety of psychostimulants for amphetamine and methamphetamine use disorders: a systematic review and meta-analysis

**DOI:** 10.1186/s13643-016-0370-x

**Published:** 2016-11-14

**Authors:** Meha Bhatt, Laura Zielinski, Lola Baker-Beal, Neera Bhatnagar, Natalia Mouravska, Phillip Laplante, Andrew Worster, Lehana Thabane, Zainab Samaan

**Affiliations:** 1 Department of Clinical Epidemiology and Biostatistics, McMaster University, 1280 Main Street W., Hamilton, ON L8S 4L8 Canada; 2 MiNDS Neuroscience Graduate Program, McMaster University, 1280 Main Street W., Hamilton, ON L8S 4L8 Canada; 3 St. George’s University of London, Cranmer Terrace, London, SW17 0RE UK; 4 Health Sciences Library, McMaster University, 1280 Main Street West, Hamilton, ON L8S 4L8 Canada; 5 Juravinski Hospital, Hamilton Health Sciences, 711 Concession Street, Hamilton, ON L8V 1C3 Canada; 6 St. Joseph’s Healthcare Hamilton, 100 West 5th Street, Hamilton, ON L8N 3K7 Canada; 7 Department of Medicine, McMaster University, 1280 Main Street West, Hamilton, ON L8S 4L8 Canada; 8 Biostatistics Unit, Centre for Evaluation of Medicine, 25 Main Street West Suite 2000, Hamilton, ON L8P 1H1 Canada; 9 System-Linked Research Unit on Health and Social Service Utilization, McMaster University, 1280 Main Street West, Hamilton, ON L8S 4L8 Canada; 10 Peter Boris Centre for Addiction Research, St. Joseph’s Healthcare Hamilton, 100 West 5th Street, Hamilton, ON L8P 3R2 Canada; 11 Population Genomics Program, Chanchlani Research Centre, McMaster University, 1280 Main St. West, Hamilton, ON L8S 4L8 Canada

**Keywords:** Amphetamine, Methamphetamine, Substance use disorder, Psychostimulants, Central nervous system stimulants

## Abstract

**Background:**

Amphetamine and methamphetamine use disorders are associated with severe health and social consequences. No pharmacological therapy has been approved for the treatment of these disorders. Psychostimulants can act as maintenance-like therapies for managing substance use among these patients.

The aim of this study is to evaluate the literature examining the efficacy and safety of psychostimulant agents for increasing abstinence and treatment retention among patients with amphetamine and methamphetamine use disorders.

**Methods:**

We searched MEDLINE, EMBASE, PsycInfo, Cochrane Central, and CINAHL from inception to August 2016. Selection of studies, data extraction, and risk of bias assessment were conducted independently by two reviewers. We conducted meta-analyses to provide a pooled summary estimate for included trials and report the review according to PRISMA guidelines.

**Results:**

We identified and selected 17 studies with 1387 participants. Outcome reporting across trials was inconsistent, and the overall quality of evidence was very low due to high risk of bias and indirectness. A meta-analysis of five trials (642 participants) found no effect of psychostimulants for end-of-study abstinence (odds ratio = 0.97, 95% confidence interval 0.65 to 1.45). Additionally, the pooled estimate from 14 studies (1184 participants) showed no effect of psychostimulants for treatment retention (odds ratio = 1.20, 95% confidence interval = 0.91 to 1.58). The incidence of serious adverse events did not differ between intervention and placebo groups based on qualitative reports from trials.

**Conclusions:**

Quantitative analyses showed no effect of psychostimulants for sustained abstinence or treatment retention. We also identified the need for more rigorous studies in this research area with clinician and patient important outcomes.

**Electronic supplementary material:**

The online version of this article (doi:10.1186/s13643-016-0370-x) contains supplementary material, which is available to authorized users.

## Background

Amphetamine-type stimulants, including methamphetamine, amphetamine, and dextroamphetamine, are highly addictive synthetic substances exhibiting increasing rates of abuse among individuals with wide-ranging sociodemographic profiles [1]. Methamphetamine use, production, and trafficking are significant problems in North America, with 61% of global methamphetamine seizures being reported within the continent in 2013 [2]. The use and abuse of amphetamine-type stimulants is a major public health concern given that initial exposure can potentially progress into dependence as rapidly as within a single week [1]. Amphetamine and methamphetamine use disorders (AMD), encompassing both abuse and dependence, are characterized by problematic patterns of the use of amphetamine-type stimulants, leading to development of tolerance and experiencing withdrawal symptoms [1]. Regular use of amphetamine-type stimulant substances can lead to adverse physical, psychological, and social consequences. Frequent methamphetamine use is associated with serious physical and psychological consequences such as increased risk of infectious diseases, cardiovascular pathology, cognitive impairment, severe dental decay, depression, psychotic disorders, and mortality [3, 4]. Methamphetamine use is also associated with social consequences such as aggression, violence, and criminal activity [4, 5]. Currently, there are no approved medications for the treatment of AMD [6, 7]. Psychosocial therapies have shown positive outcomes, but results are inconsistent for treatment retention and many trials show no reduction in substance use at follow-up [8, 9]. A previous research has called for future work to determine which medications are effective for relief of withdrawal symptoms and to be used in combination with psychosocial treatments [9]. Given the medical and social costs of AMD, it is necessary to identify an effective harm reduction therapy.

A number of randomized controlled trials (RCTs) have investigated the effectiveness of psychostimulants as harm reduction therapy for AMD. Psychostimulant agents are generally dopamine (DA) agonists that increase synaptic concentrations of DA with similar mechanisms of action as abused amphetamines and methamphetamines [10]. Abused amphetamines and methamphetamines increase synaptic concentrations of monoaminergic neurotransmitters, including DA [10]. The expectation is for psychostimulants to act as maintenance-type therapy for patients with AMD to relieve withdrawal symptoms and drug cravings, subsequently leading to reduced stimulant use. Previous reviews have been published [6, 7, 10, 11], with only the most recent review by Pérez–Mañá et al. [7] conducting a meta-analysis. Pérez–Mañá and colleagues [7] found no effect of psychostimulants for AMD. However, a number of trials have been conducted with psychostimulants since the completion of this review. Our systematic review will broaden eligibility criteria by including adolescent and adult studies, and conducting subgroup analyses by age group and frequency of substance use, which may have an effect on treatment outcome [12, 13]. Pérez–Mañá et al. [7] reported that the quality of evidence in this area was very low, indicating that the new trials published since then may have a substantial impact on the treatment outcome. Additionally, due to the small sample size of individual trials, an updated review is necessary to summarize the evidence.

The objective of the present review is to summarize the efficacy and safety of psychostimulant medications for the treatment of AMD in adolescents and adults. Specifically, we aim to summarize the current literature examining the efficacy of psychostimulant medications for (1) sustaining abstinence from illicit amphetamines and methamphetamines and (2) retention in treatment by conducting risk of bias and quality assessment of included trials, and computing quantitative pooled summary estimates. Secondary objectives of the review are to consider the potential modifiers of psychostimulant treatment efficacy and summarize the risk of serious adverse events (SAEs) related to psychostimulant therapy, as reported by the included studies.

## Methods

The following methods were established a priori in an unpublished protocol that is available upon request from the authors. The review was submitted to PROSPERO for registration but did not meet eligibility criteria at the time of submission. The review is reported in accordance with the Preferred Reporting Items for Systematic Reviews and Meta-Analyses (PRISMA) guidelines [14] and an additional file shows the PRISMA checklist (see Additional file 1).

### Data sources

The following databases were searched from inception until August 2016: MEDLINE, EMBASE, PsycINFO, Cochrane Central Library, and Cumulative Index to Nursing and Allied Health Literature (CINAHL). A broad search was employed in order to capture all relevant citations. An additional file outlines the search strategy, developed by the first author (M.B.) in collaboration with an expert health sciences librarian (N.B.) (Additional file 2). Furthermore, reference lists of past systematic reviews and included trials were manually searched for relevant studies. ClinicalTrials.gov was searched as a source for unpublished and ongoing trials.

### Inclusion and exclusion criteria

The search was limited to studies with human participants. However, all databases were searched without restrictions on language or date of publication. We included RCTs comparing the use of any psychostimulant medication to an inactive control group (i.e., placebo) for the treatment of AMD. We excluded all study designs that were not RCTs, including non-randomized clinical trials and quasi-randomized studies. Participants included adolescents and adults ≥14 years of age and diagnosed with amphetamine or methamphetamine use disorder (including those with abuse or dependence), according to the Diagnostic and Statistical Manual of Mental Disorders (DSM) criteria. We included studies that used clinician-administered or self-reported validated instruments for diagnosis of AMD. We also included studies of participants diagnosed with DSM-5 criteria stimulant use disorder (including cocaine, amphetamine, and methamphetamine) and extracted relevant data about patients with only AMD. Trials of participants with cocaine use disorder alone were excluded. Studies examining the use of psychostimulants or central nervous system stimulants for the treatment of AMD were included in the review. Since “psychostimulant” agents represent a pharmacological effect rather than class of medications, a comprehensive list of these agents is not available. Instead, psychostimulants are dispersed over various pharmacological groups based on their indication. We utilized an approach described by Castells et al. [15] and Pérez–Mañá et al. [7] in previous reviews of stimulant use disorders to obtain a comprehensive list of psychostimulants. We identified drugs with psychostimulant modes of action from the Anatomical Therapeutic Chemical Classification [16] and the American Hospital Formulary Service Pharmacologic–Therapeutic Classification System [17] and included in the search strategy. The strategy included psychoanaleptics with mild stimulant effects (bupropion, modafinil) as well as classical stimulants (dextroamphetamine, methylphenidate, and dexmethylphenidate). Trials managing withdrawal rather than dependence were excluded. Trials were excluded if they assessed non-substance use outcomes, such as cognitive and neurological outcomes, after in-lab administration of amphetamines or methamphetamine.

### Data collection and extraction

Two authors independently screened titles and abstracts of all retrieved citations, excluding studies that failed to meet eligibility criteria. Following title and abstract screening, two authors independently reviewed full-texts of relevant studies. Studies that satisfied all eligibility criteria were included for data extraction. We used Covidence systematic review software (Veritas Health Innovation, Melbourne, Australia) for screening and full-text review. We resolved disagreements during the screening process by discussion to consensus. If they remained unresolved, a senior author was consulted to determine study eligibility. Two authors extracted data from included trials using a pilot tested data extraction form. An additional file includes the completed form with details extracted from individual studies (see Additional file 3), which was later exported into Review Manager Version 5.3 (The Cochrane Collaboration) for statistical analysis.

### Risk of bias and quality assessment

Two raters independently assessed risk of bias of included trials using the Cochrane Collaboration’s risk of bias tool [18]. Scores of *high*, *low*, or *unclear* risk of bias were assigned to trial factors including random sequence generation, allocation concealment, blinding of participants, personnel and outcome adjudicators, incomplete outcome assessment, selective reporting, and other sources of bias. We assessed publication bias visually by generating a funnel plot for primary outcomes. We used the risk of bias assessment to support conclusions regarding the overall quality of evidence in the review. We applied the Grading of Recommendations, Assessment, Development, and Evaluation (GRADE) framework for systematic reviews to the included trials to assign an overall outcome-specific rating for risk of bias, inconsistency, indirectness, imprecision, and publication bias [19]. We used GRADE Pro GDT software (http://gdt.guidelinedevelopment.org/app/) to create evidence and summary of findings tables.

### Data synthesis

We measured inter-rater agreement using the unweighted kappa statistic for full text review and risk of bias assessment and conducted meta-analyses using Review Manager Software Version 5.3 (The Cochrane Collaboration, London, UK).

The primary outcomes of this review were (1) abstinence from illicit amphetamines and methamphetamines and (2) retention in treatment. Abstinence was measured as the proportion of participants having substance-free urine tests in intervention and control groups. The longest substance-free period of time assessed across multiple studies was used to determine sustained abstinence. We measured treatment retention, or completion of treatment, as the proportion of individuals remaining in treatment at the end of the trial. The secondary outcome was the incidence of SAEs for different psychostimulant interventions in comparison with placebo. SAEs were defined as any medical or psychiatric event causing hospitalization or dropout from the study.

We summarized individual study results qualitatively and conducted meta-analyses to obtain pooled summary estimates (odds ratios), when interventions and outcomes were comparable between trials. We pooled studies broadly and conducted predetermined subgroup analyses to explore heterogeneity. We measured heterogeneity using the inconsistency index (*I* ^2^) for each meta-analysis, with *I* ^2^ >40% representing substantial heterogeneity. We applied the random-effects model with inverse variance methods for meta-analysis, as it accounts for within and between-study variability and considerable variability is expected between included trials [20]. We analyzed all data according to the intention-to-treat principle with the number of participants randomized being used as the denominator. If multiple trials reported overlapping of study participants, only the trial with the larger sample was included in the meta-analysis so as to not to violate the assumption of independence.

### Subgroup analyses

We conducted the following a priori planned subgroup analyses:

1. A subgroup analysis based on participant diagnosis of amphetamine or methamphetamine use disorder due to differences between individuals with the different substance dependencies. It was hypothesized that the effect would favor participants with amphetamine use disorder due to similarity of psychostimulant effect to that of abused amphetamines.
2. A subgroup analysis comparison of different psychostimulant interventions studied. It was expected that dexamphetamine would have a favorable effect compared to other psychostimulant interventions due to similarity of structure to that of abused amphetamine substances.
3. A subgroup analysis based on frequency of amphetamine and methamphetamine use in the past month (number of days). Less than daily use of amphetamines or methamphetamines was considered low frequency relative to studies including any frequency of use. We expected that studies examining lower baseline frequency of substance use would demonstrate greater treatment effect [12, 13].
4. A subgroup analysis based on age of participants, by comparing adolescent or adult studies. Due to challenges in treatment retention and overall adherence to treatment among adolescent populations, it was hypothesized that adult studies would show greater treatment effect.
5. A subgroup analysis on duration of treatment based on length ≤12 or >12 weeks of treatment. It was expected that studies with >12 weeks of treatment would show favorable effect, based on previous substance use intervention studies finding similar results for treatment programs with longer duration.

We planned an additional subgroup analysis to compare dosage of psychostimulant interventions but could not conduct due to similar doses between studies examining the same medications.

### Sensitivity analysis

We conducted sensitivity analyses by removing studies with high risk of bias for each item on the Cochrane risk of bias tool in order to evaluate the robustness of the summary estimates for each outcome. We also conducted a sensitivity analysis by removing studies conducted within specific high-risk populations (participants with attention-deficit hyperactivity disorder, criminal offenders, and men who have sex with men).

## Results

### Description of studies

**Fig. 1:**
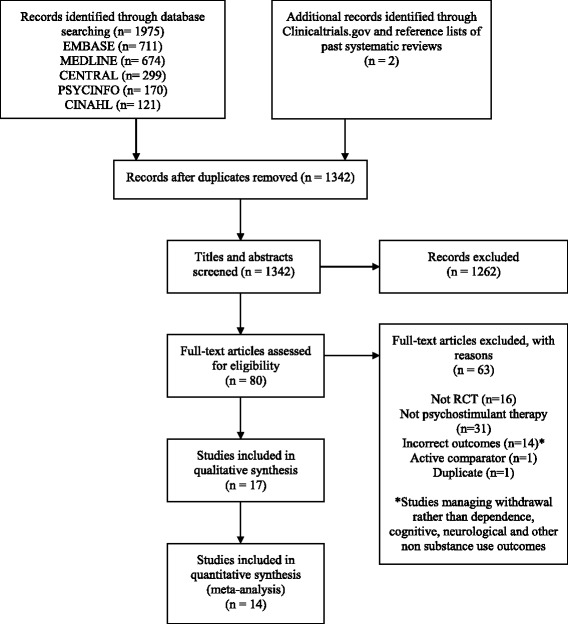
PRISMA flow diagram for the study selection process

Following screening of 1251 unique citations for eligibility, 17 studies were included for data extraction and analysis in the review (Fig. 1). The unweighted kappa for full-text review for two independent reviewers was 0.84. We also calculated inter-rater agreement for the risk of bias assessment, yielding a kappa statistic of 0.83, indicating excellent levels of agreement between raters, as per Cochrane Handbook cutoffs.

Seventeen studies were included in the systematic review, all of which were parallel design RCTs published in a peer-reviewed journal [12, 13, 21–35]. Table 1 displays individual study characteristics of included trials and summarizes substance use outcomes for all trials, including those that are not included in the quantitative analysis.

**Table 1:** Individual study characteristics (17 included studies)

Author, year and country	Diagnosis/sample details	Study length	Intervention/dose	Number of participants (% male)	Mean age by treatment group (years), SD	Summary of substance use outcomes
Anderson, A 2012 USA [35]	MA dependence/adults with any frequency of use	16 weeks	Modafinil/200 or 400 mg once daily on awakening	210 (59.1% male)	Modafinil 200 mg 37.6 (8.9) Modafinil 400 mg 40 (8.4) Placebo 39.4 (8.6)	No significant difference in MA non-use weeks (*p* = 0.53, GEE), MA non-use days (*p* = 0.63, overall Kruskal–Wallis) or terminal abstinence (Fisher’s exact *p* = 0.84) between modafinil groups and placebo
Anderson, A 2015 USA [12]	MA dependence/low frequency users (≤29 of past 30 days)	12 weeks	Bupropion/150 mg twice daily	204 (65% male)	Total sample 39.3 (NR)	Treatment success, defined as ≥2 negative urines in weeks 11 and 12, was achieved by 14% (14/100) of the bupropion group and 19% (20/104) of the placebo group (chi-square, *p* = 0.32)
Das, M 2010 USA [21]	MA dependence/among men having sex with men	12 weeks	Bupropion/150 mg 1 pill every morning for 1 week, then 2 150 mg pills every morning thereafter	30 (100% male)	Bupropion 38.1 (2) Placebo 33.3 (3)	Reductions in meth-metabolite-positive urines were similar in the bupropion and placebo groups (normal-logistic model, *p* = 0.63)
Elkashef, A 2008 USA [22]	MA dependence/adults with any frequency of use	18 weeks	Bupropion/150 mg once daily for 3 days, then increased to 300 mg daily (1 tablet twice a day) for 11 weeks	151 (67% male)	Bupropion 36.2 (9.2) Placebo 35.7 (8.4)	No significant improvement for percentage of participants with MA-free study weeks in the bupropion group (week 1 = 25%; week 12 = 54%) compared to placebo (week 1 = 29%; week 12 = 44%) (GEE, *p* = 0.09)
Galloway, G 2011 USA [23]	MA dependence/adults with any frequency of use	8 weeks	Dextroamphetamine/60 mg daily—single dose on the first day and as 2 equally divided doses on subsequent days	60 (56.7% male)	Dextroamphetamine 37 (7.2) Placebo 37.5 (7.2)	Out of 16 urine tests over the 8-week trial period, the dextroamphetamine group had 2.9 ± 4.3 MA-negative results and the placebo group had 3.2 ± 5.0 MA-negative results (Mann–Whitney *U* test: *W* = 441, *p* = 0.894)
Heinzerling, K 2010 USA [24]	MA dependence/adults with any frequency of use	14 weeks	Modafinil/200 mg per day (taken in the morning) for the first 3 days of the study, followed by an increase to 400 mg per day (in the morning)	71 (70.4% male)	Modafinil 39.1 (11.1) Placebo 37.8 (10.1)	No significant association between treatment group assignment and the probability of providing MA-free urine drug screens across the treatment period (OR = 0.78, 95% CI 0.39–1.56, *p* = 0.49 for modafinil relative to placebo)
Heinzerling, K 2013 USA [33]	MA abuse or dependence/adolescent low frequency users (≤18 of past 30 days)	8 weeks	Bupropion/150 mg twice daily	19 (47.4% male)	Bupropion 17.5 (1.6) Placebo 17.7 (1.1)	Mean number of twice weekly MA-negative urine screens in bupropion group = 5.0 and placebo group = 8.9 (*p* = 0.043)
Heinzerling, K 2014 USA [13]	MA dependence/low frequency users (≤29 of past 30 days)	16 weeks	Bupropion/150 mg once daily for 3 days, then 150 mg twice daily	84 (80.9% male)	Bupropion 38.6 (10.1) Placebo 38.1 (10.3)	No significant difference in the proportion of participants with MA abstinence during weeks 11 and 12, for bupropion = 12/41 and placebo = 6/43 (*p* = 0.087)
Konstenius, M 2010 Sweden [25]	Amphetamine dependence/among individuals with ADHD	13 weeks	Methylphenidate/18 mg starting dose titrated over period of 10 days to the maximum dose of 72 mg	24 (75% male)	Methylphenidate 34.6 (10.1) Placebo 39.7 (9.8)	No significant difference in proportion of positive urine screens during the study between methylphenidate (mean = 10.6, SD = 8.8) and placebo groups (mean = 8.6, SD = 7.8) (*p* = 0.472)
Konstenius, M 2014 Sweden [26]	Amphetamine dependence/among incarcerated individuals with ADHD	24 weeks	Methylphenidate/18 mg starting dose titrated over a period of 19 days (with 36 mg increments every 3 days), to a maximum dose of 180 mg/day	54 (100% male)	Methylphenidate 41 (7.5) Placebo 42 (11.7)	Significant difference in proportion of drug-negative urines in methylphenidate group (MD = 23%, *n* = 27) compared to placebo group (MD = 16%, *n* = 27) *p* = 0.047
Ling, W 2014 USA [27]	MA dependence/adults with any frequency of use	14 weeks	Methylphenidate/18 mg daily for week 1, 36 mg for week 2 and 54 mg for weeks 3–10	110 (81.8% male)	Methylphenidate 38.7 (9.8) Placebo 39.5 (10.4)	Methylphenidate group was less likely to be MA positive compared to placebo group at week 14 (OR = 0.18, *p* = 0.025)
Longo, M 2009 Australia [29]	MA dependence/adults with any frequency of use	12 weeks	Dexamphetamine/20 mg/day starting dose increased by 10 mg daily as required until stabilized or to a maximum of 110 mg/day (stabilized over 14 days)	49 (61.2% male)	Dexamphetamine 31.9 (4.5) Placebo 31.9 (5.6)	Significant decrease of MA concentration in hair for both groups (*p* < 0.0001) but no significant difference between groups (*p* value not provided)
Miles, SW 2013 Finland and New Zealand [28]	Amphetamine or MA dependence/adults with any frequency of use	22 weeks	Methylphenidate/18 mg/day for the first week, 36 mg daily for the second week and 54 mg daily thereafter until the end of week 22	78 (62.8% male)	Methylphenidate 38.9 (9.2) Placebo 34 (8.5)	No significant difference in mean percentage of positive urine tests over the course of the study between methylphenidate (mean = 89%, SD = 19) and placebo (mean = 90%, SD = 14) groups (*p* = 0.89)
Rezaei, F 2015 Iran [31]	MA dependence/adults with any frequency of use	10 weeks	Methylphenidate/18 mg/day during the first week and 36 mg/day during the second week and then received 54 mg/day for the remaining 8 weeks	56 (73.2% male)	Methylphenidate 35.6 (6.9) Placebo 34.7 (9.1)	Methylphenidate group had significantly less MA-positive urine tests compared to placebo at week 10 (*p* = 0.03)
Shearer, J 2009 Australia [34]	MA dependence/regular users (2–3 days of use per week or more)	22 weeks	Modafinil/200 mg/day	80 (62.5% male)	Modafinil 35.8 (6.9) Placebo 36.1 (9.1)	No significant difference in proportion of stimulant-positive weekly urine drug screens between groups (chi-square = 17.10, *p* = 0.07)
Shoptaw, S 2008 USA [30]	MA dependence/adults with any frequency of use	12 weeks	Bupropion/150 mg per day for days 1–3, followed by an increase to 300 mg per day (one 150 mg capsule taken twice daily) until week 12	73 (64.4% male)	Bupropion 34.6 (10.6) Placebo 34.6 (10.0)	No significant difference between treatment groups in the mean MA-free urine screens or the probability of achieving a MA-free week in a GEE model (chi-square = 0.004, degrees of freedom = 71, *p* = 0.95)
Tiihonen, J 2007 Finland [32]	Amphetamine or MA dependence/among intravenous users	20 weeks	Methylphenidate/18 mg/day for the first week, 36 mg/day for the second week, and 54 mg/day thereafter	34 (70.6% male)	Methylphenidate 35.1 (7.9) Placebo 40 (10.1)	Significantly fewer positive urine samples in methylphenidate group compared to placebo group (OR = 0.42, 95% CI = 0.24–0.72; *z* = −3.14, *p* = 0.002)

All studies used an inactive placebo pill of the same dosage as the intervention for the control group. The substance use outcomes are summarized as they are reported in the individual studies

*MA* methamphetamine, *SD* standard deviation, *NR* not reported, *GEE* generalized estimating equations, *OR* odds ratio, *CI* confidence interval, *MD* mean difference

Four different psychostimulant therapies were examined in the included studies—modafinil [24, 34, 35], bupropion [12, 13, 21, 22, 30, 33], methylphenidate [25–28, 31, 32], and dexamphetamine/dextroamphetamine [23, 29].

Most studies enrolled participants with DSM–IV methamphetamine dependence, while two studies recruited participants with both amphetamine and methamphetamine dependence [28, 32]. Furthermore, two other studies included participants with a diagnosis of amphetamine dependence alone and comorbid attention-deficit hyperactivity disorder (ADHD) [25, 26]. There was only one trial conducted in adolescents [33], while the remaining trials were within adult samples, 18–65 years of age. The adolescent study was conducted among participants with ≤18 days of methamphetamine use in the month prior to baseline interview [33]. Two other studies were conducted among low-frequency adult methamphetamine users (≤29 days in the month prior to baseline interview) [12, 13]. Men comprised >60% of the study sample in many studies, and two studies included only men [21, 26]. Among those studies, Das et al. [21] specifically included men who have sex with men, a population at increased risk of methamphetamine use and abuse. A number of studies excluded individuals diagnosed with comorbid substance use disorders [13, 24–26, 28–30, 33, 35].

The majority of trials administered the psychostimulant intervention for 8–12 weeks, while few studies had longer treatment duration (20–24 weeks) [25, 28, 32]. Doses of medications were relatively consistent within psychostimulant type: however, the dose varied between different types of psychostimulants. All studies investigating modafinil administered doses of 200 mg, with the exception of study by Anderson et al. [35] having a second treatment group receiving 400 mg of modafinil. For the Anderson [35] trial, the number of events from the 200 and 400 mg treatment groups was combined in the meta-analysis and compared to the placebo group. Bupropion was consistently given in doses of 150 mg twice daily, and the maximum dose of methylphenidate ranged from 54 to 180 mg between studies. There were two trials of dextroamphetamine/dexamphetamine—both stabilizing participants at different doses. All trials were placebo-controlled, although most trials did not report the inactive substance in the placebo capsule. Two trials used lactose [23, 27] and one used gelatin [32] as the inactive substance in the placebo group.

### Risk of bias assessment

**Fig. 2:**
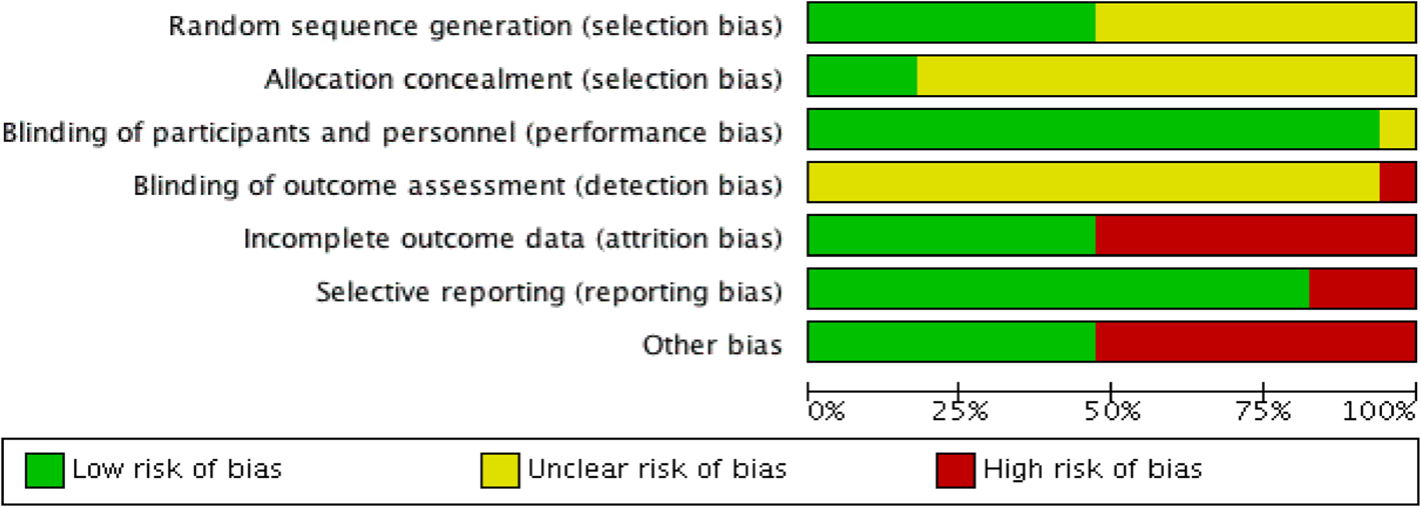
Summary graph of author judgments for each risk of bias criteria

**Fig. 3:**
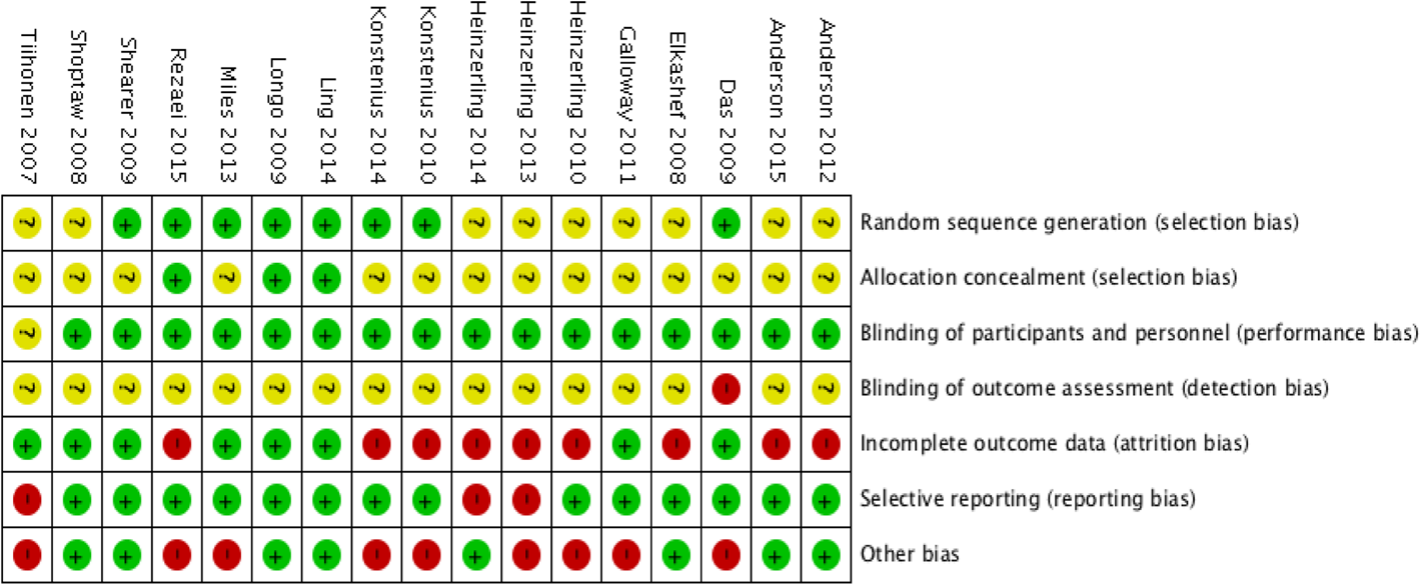
Risk of bias assessment based on author judgment for individual studies

Figures 2 and 3 display a summary of the risk of bias assessment, conducted in duplicate by the review authors using the Cochrane risk of bias tool. A number of studies had unclear or high risk for at least one methodological criterion. Random sequence generation was adequately reported in less than half of the trials, and over 75% of the trials did not report allocation concealment adequately. While 16 of the 17 trials implemented blinding of participants and study personnel, all trials had unclear or high risk of bias for blinding of outcome assessment.

Attrition was a major drawback in most trials, with >50% of participants dropping out before study completion. However, the majority of trials conducted an intention-to-treat analysis and had similar proportions of dropout in intervention and placebo groups. High risk of bias for incomplete outcome data was still an issue for 50% of the trials when reasons for dropouts were not stated, and it could not be determined whether reasons for dropout were similar for intervention and control groups. Risk of bias due to selective outcome reporting was primarily *low*, although study outcomes were reported inconsistently between trials.

All studies required written informed consent from participants and reported approval from an ethics review board. The majority of trials were not industry funded, but some study authors reported a conflict of interest. Other bias resulted from significant conflicts of interest (as judged by the rater), imbalanced randomization judged to have potential impact on the outcome, and underpowered based on presented sample size/power calculation. There were also a few studies with 17 or less participants in each treatment arm, which were likely biased towards type II errors due to small sample sizes [21, 25, 32, 33].

### Meta-analyses: efficacy of psychostimulants for abstinence from illicit amphetamine and methamphetamine

Five out of 17 included studies were included in the primary meta-analysis for abstinence from illicit amphetamines or methamphetamines [12, 13, 24, 30, 35]. All of the five studies included in the meta-analysis included participants with methamphetamine dependence (regular or low frequency of use) and measured “terminal abstinence,” defined as methamphetamine-free urine screens during the final 2 weeks of the trial. The remaining studies were excluded from the meta-analysis because outcomes were not reported consistently, and the studies lacked sufficient information to determine the number of participants with sustained abstinence in intervention and control groups.

**Fig. 4:**
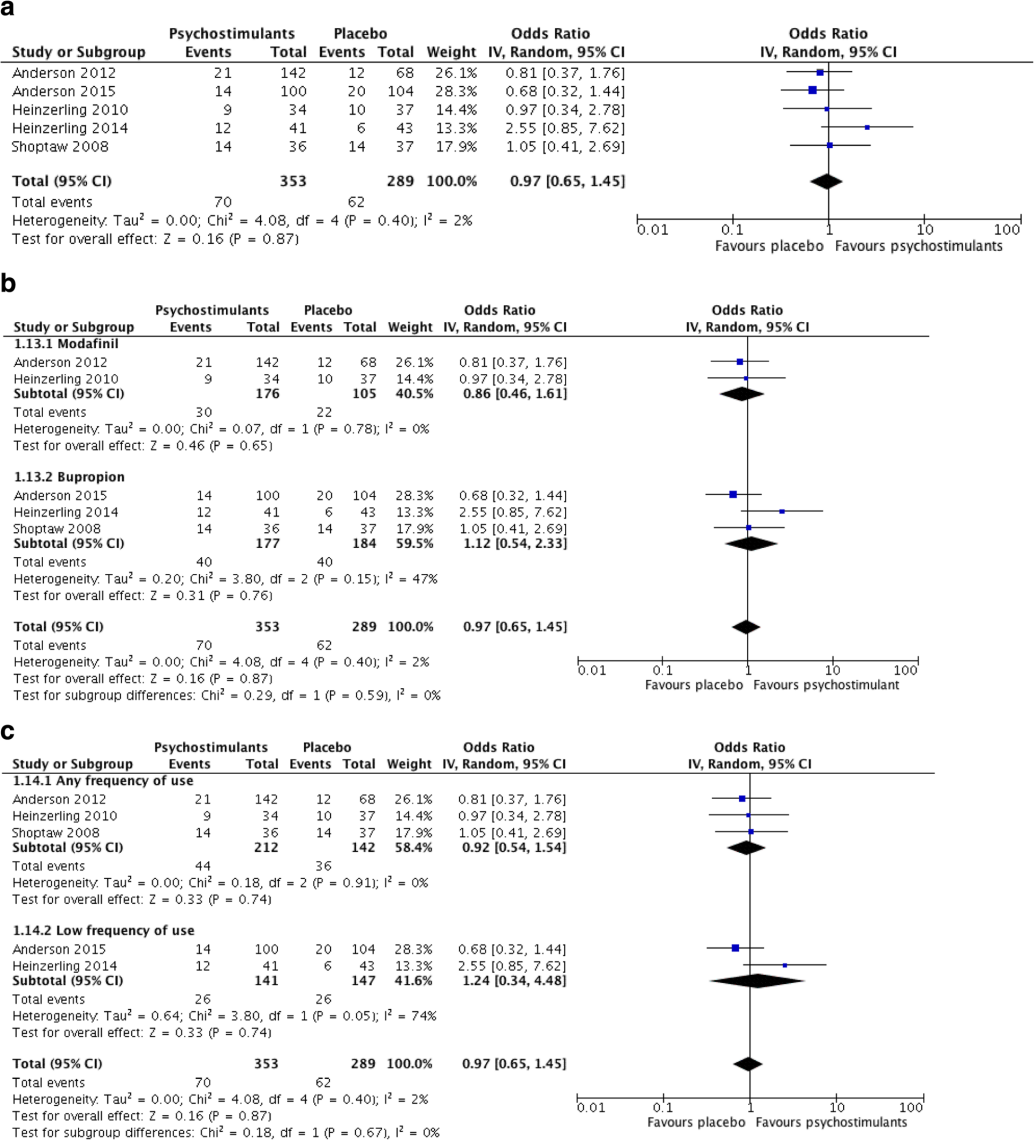
**a** Forest plot for efficacy of psychostimulants on abstinence from illicit amphetamines or methamphetamines (measured by urinalysis). **b** Subgroup analysis by psychostimulant medication on abstinence from illicit amphetamines or methamphetamines. **c** Subgroup analysis by frequency of substance use on abstinence from illicit amphetamines or methamphetamines

Figure 4a shows the pooled summary estimate, based on five studies and a total sample size of 642 individuals. Of these participants, 353 received psychostimulant therapy and 289 received placebo. The pooled odds ratio for sustained abstinence was 0.97 (95% confidence interval 0.65 to 1.45, *p* value = 0.87), representing no significant effect of psychostimulants on abstinence from methamphetamines. This summary estimate was associated with low statistical heterogeneity, as indicated by an *I* ^2^ of 2% (chi-square = 4.08, degrees of freedom = 4, *p* value = 0.40). Sources of heterogeneity were explored by conducting a priori specified subgroup analyses.

### Subgroup analyses

Due to inclusion of five trials in the meta-analysis, we were unable to conduct subgroup analyses based on age (adolescent or adult), substance use disorder (amphetamine or methamphetamine use disorder), or treatment duration. The first subgroup analysis was conducted based on psychostimulant intervention. The modafinil group was associated with no heterogeneity (*I* ^2^ = 0%, *p* value = 0.78), while the bupropion group showed substantial heterogeneity (*I* ^2^ = 47%, *p* value = 0.15). However, both modafinil (OR = 0.86, 95% CI 0.46 to 1.61, *p* value = 0.65) and bupropion (OR = 1.12, 95% CI 0.54 to 2.33, *p* value = 0.76) showed no effect for sustained abstinence (Fig. 4b). The test for subgroup differences was not significant (*I* ^2^ = 0%, chi-square = 0.29, degrees of freedom = 1, *p* value = 0.59).

The second subgroup analysis was conducted for frequency of substance use and showed high heterogeneity among studies with low frequency (i.e., non-daily) substance users (*I* ^2^ = 74%, *p* value = 0.05). Figure 4c shows the forest plot. Studies with unspecified frequency of use were associated with no heterogeneity (*I* ^2^ = 0%, *p* value = 0.91). However, both low frequency (OR = 1.24, 95% CI 0.34 to 4.48, *p* value = 0.74) and unspecified frequency (OR = 0.92, 95% CI 0.54 to 1.54, *p* value = 0.74) substance use groups showed no effect for abstinence. The test for subgroup differences was again associated with no heterogeneity but remained non-significant (*I* ^2^ = 0%, chi-square = 0.18, degrees of freedom = 1, *p* value = 0.67).

### Meta-analyses: efficacy of psychostimulants for retention in treatment

**Fig. 5:**
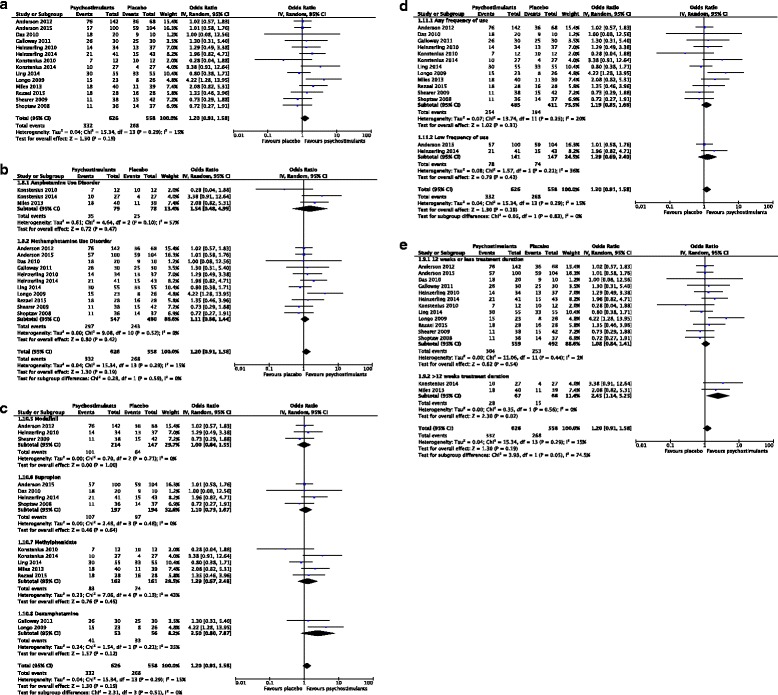
**a** Forest plot for efficacy of psychostimulants on retention in treatment. **b** Subgroup analysis by substance use disorder on retention in treatment. **c** Subgroup analysis by psychostimulant medication on retention in treatment. **d** Subgroup analysis by frequency of substance use on retention in treatment. **e** Subgroup analysis by treatment duration on retention in treatment

Fourteen studies were included in the meta-analysis for the outcome of retention in treatment, defined as the proportion of participants that completed the study (Fig. 5a). Two studies were excluded because information about dropouts was insufficient to determine the number of paticipants retained in treatment [32, 33], and a study by Elkashef et al. [22] was excluded due to an overlapping sample with Anderson et al. [12]. The pooled summary estimate was based on a collective 1184 participants, of whom 626 received psychostimulant interventions and 558 received placebo. The pooled OR (1.20) was non-significant (95% CI 0.91 to 1.58) and was associated with low heterogeneity (*I* ^2^ = 15%, chi-square = 15.34, degrees of freedom = 13, *p* value = 0.29). We explored sources of heterogeneity by prespecified subgroup analyses, but did not conduct a subgroup analysis of age because the adolescent study was excluded from the meta-analysis due to insufficient outcome data [33].

### Subgroup analyses

The first subgroup analysis was based upon study inclusion of participants with amphetamine use disorder and methamphetamine use disorder (Fig. 5b). The test for subgroup differences was not statistically significant (*I* ^2^ = 0%, chi-square = 0.28, degrees of freedom = 1, *p* value = 0.59). The amphetamine use disorder subgroup was associated with substantial heterogeneity (*I* ^2^ = 57%) based on the cutoff of high heterogeneity at >40%, and the pooled OR of 1.54 was not statistically significant (95% CI 0.48 to 4.99, *p* value = 0.47). The methamphetamine use disorder subgroup was associated with no heterogeneity (*I* ^2^ = 0%), and a non-significant overall OR of 1.11 (95% CI 0.86 to 1.44, *p* value = 0.42). The test for subgroup differences was associated with no heterogeneity but was not significant (*p* value = 0.59).

We conducted a second subgroup analysis by specific type of psychostimulant medication, varying between two to six studies within the subgroups (Fig. 5c). A test for differences in effect between the aforementioned subgroups was not significant (chi-square = 2.31, degrees of freedom = 3, *p* value = 0.51), but associated with no heterogeneity (*I* ^2^ = 0%). No subgroups of psychostimulants showed a significant OR for treatment retention, and only the methylphenidate subgroup was associated with substantial heterogeneity (*I* ^2^ = 43%, *p* value = 0.13). There was a trend in both studies of dexamphetamine for favorable effects of psychostimulants; however, there was no significant pooled effect (OR = 2.50, 95% CI 0.80 to 7.87, *p* value = 0.12) and the 95% confidence interval was wide.

We conducted the third subgroup analysis (Fig. 5d) based upon frequency of substance use disorder. As previously defined, low frequency of substance use was characterized as substance use ≤29 days in the past month (i.e., non-daily substance use) and was compared to studies including participants with unspecified frequency of substance use. None of the subgroups showed a statistically significant pooled estimate for treatment retention; however, the low frequency subgroup only included two studies. The test for subgroup differences showed no heterogeneity and was not significant (chi-square = 0.05, degrees of freedom = 1, *p* value = 0.83).

The final subgroup analysis for this outcome was based on treatment duration, categorized as ≤12 weeks or >12 weeks (Fig. 5e). The test for subgroup differences was borderline significant and associated with substantial heterogeneity (*I* ^2^ = 74.5%, chi-square = 3.93, degrees of freedom = 1, *p* value = 0.05). The subgroup of studies with ≤12 weeks of treatment did not find a significant pooled OR. The pooled estimate of two studies >12 weeks was significant and associated with no heterogeneity. Results showed twice the increased odds of retention in treatment >12 weeks for the psychostimulant group with an OR = 2.45 (95% CI 1.14 to 5.25, *p* value = 0.02).

### Psychostimulant safety: adverse events

Three of the 17 studies utilized a standardized instrument for investigating differences in adverse events between psychostimulant and placebo groups [23, 28, 34], while the remaining studies reported observed adverse events. Miles et al. [28] measured adverse events using the Udvalg Kliniske Undersogelser and found no differences between methylphenidate and placebo arms. Shearer et al. [34] reported no statistically significant univariate differences in mild, moderate, or serious adverse events between modafinil and placebo groups. Similarly, Galloway et al. [23] found no differences in reported adverse events between dextroamphetamine and placebo groups. Across all studies, mild adverse events differed by psychostimulant medication but these mild symptoms were not associated with dropout or discontinuation of medication in most studies.

While a number of studies reported SAEs including hospitalization due to suicidal ideation, seizures, pneumonia, and others, no study stated that the SAEs or dropouts were associated with active study medication. However, there was high inconsistency in reporting of adverse events, and it was often unclear whether SAEs resulted in complete discontinuation from the study. From the available information, we found there were 8 dropouts from modafinil groups [24, 34, 35], 11 from bupropion [12, 13, 22, 30], and 7 from methylphenidate [27] (26 total) and a total of 11 from placebo groups [13, 22, 24, 26–28, 30].

### Sensitivity analyses

We conducted sensitivity analyses by removing studies within sub-populations one at a time. Das (2010)  included a sample of men who have sex with men, Konstenius et al. [25] comprising of adults with comorbid ADHD, and Konstenius et al. [26] of men who are criminal offenders with ADHD were removed, but there were no differences in the results of the meta-analyses for the primary outcomes. Additionally, the pooled estimate did not change significantly for either primary outcome when we removed studies with high risk of bias for “selective outcome reporting” and “incomplete outcome data” one at a time.

### Publication bias

**Fig. 6:**
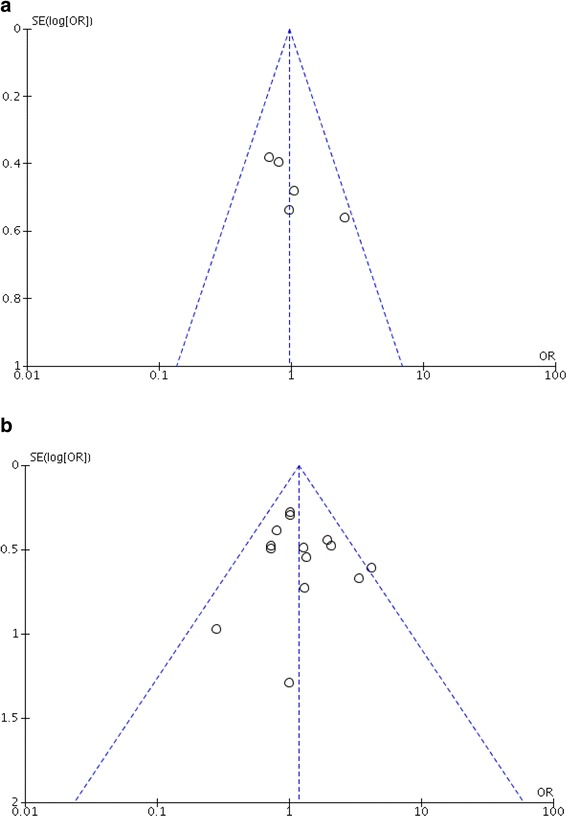
**a** Funnel plot for abstinence from illicit substances. **b** Funnel plot for retention in treatment

Figure 6a, b show funnel plots assessing publication bias for treatment retention. Figure 6a generates using the five studies from the meta-analysis assessing abstinence from amphetamine and methamphetamine use. The left side of the plot favors placebo, and the right side of the plot favors psychostimulants with regard to increased odds of abstinence from illicit stimulants. There is no evidence of asymmetry in the funnel plot based on five of the 17 included studies. However, it is expected that the plot will remain symmetrical after addition of the remaining studies since a number of studies with negative results have been published. Figure 6b assesses publication bias for treatment retention based on 14 included studies. There is likely no publication bias since the plot shows point estimates in the left lower quadrant, indicating that that negative findings of psychostimulant treatment effect have been published regardless of the study size.

## Discussion

This systematic review included 17 trials investigating the efficacy and safety of psychostimulants in patients diagnosed with AMD. Five out of 17 studies were included in the meta-analysis for the primary outcome of sustained abstinence. Studies included in the meta-analysis for abstinence all investigated psychostimulants for methamphetamine dependence, and no effect was found for this outcome. Subgroup analyses of abstinence also found no effect. For the outcome of treatment retention, 14 studies were included in the meta-analysis and the odds ratio was pooled. Overall, we found no significant effect of psychostimulants on treatment retention. However, the subgroup analyses for treatment retention demonstrated favorable effects for treatments with longer duration (>12 weeks). The literature has shown that patients enrolled in longer duration general substance use treatment programs tend to have more successful treatment outcomes [36]. These results are interesting because they indicate that participants in longer duration studies are at increased odds of retention until the end of treatment: however, this finding is based on a subgroup of two studies and cannot be generalized for all samples [26, 28]. It is possible that longer duration treatment may also show increased effectiveness for substance use outcomes such as abstinence from amphetamines and methamphetamine, which is important to examine with further research. However, this finding of higher retention may also be due to factors such as standard of care, including improved therapeutic alliance or counseling community, in longer duration studies. As well, continued participation may have occurred due to expectation of early parole or fear of extended incarceration in the sample of criminal offenders recruited from prisons [26]. In the remaining subgroup analyses, we found no significant effects, although the direction of effects was consistent with our hypotheses. Other reviews have reported modafinil to be effective in reducing amphetamine and methamphetamine use [6, 9]; however, these reviews did not quantitatively summarize the results. The present review found no effect of modafinil or bupropion on sustained abstinence from substances in the meta-analysis.

There was a high level of inconsistency in reporting adverse events and safety outcomes among included studies; therefore, the overall observed number of SAEs was determined for each psychostimulant intervention and placebo groups. There were no clear differences in reported adverse events based on raw numbers between intervention and placebo groups. However, adverse events may have been impacted by imperfect collection and reporting methods, since only three of 17 studies used standardized instruments to collect adverse event data [23, 28, 34]. Development of adverse event reporting standards for RCT’s in this area and transparent reporting of the methods used to ascertain whether SAEs were associated with study medication is necessary. This is especially important due to the increasing number of trials conducted in recent years, which have shown no effect for reduction of substance use [12, 13, 28, 33]. While it remains challenging to conduct clinical trials in the field of substance use disorders, improving the quality of future trials is important to detect a true treatment effect that is not significantly impacted by methodological limitations.

Previous reviews for pharmacological interventions have been conducted for general stimulant dependence [6, 9–11, 37]; however, most reviews provide narrative summaries of results whereas only the most recent review is specific to AMD patients and conducts meta-analyses [7]. Since the publication of the recent review by Pérez–Mañá et al. [7], a reasonable number of new trials examining psychostimulants have been conducted [12, 13, 26–28, 31]. Though new trials in this area are being conducted, the methodology and reporting of outcomes remains largely unchanged, making it challenging to utilize trial results for quantitative analysis in systematic reviews. Substance use outcomes across the RCTs include the proportion of negative urine screens within a group, mean methamphetamine non-use days or weeks, longest period of time abstinent in days or weeks, mean number of negative urine screens, and number of participants with terminal or end-of-trial abstinence. Lack of uniformity in outcome reporting among trials investigating pharmacological therapies for AMD has resulted in several recent trials reporting group level outcomes such as overall proportion of negative urine screens in each trial arm [21, 26, 28, 31, 32, 34]. Group level outcomes make it difficult to ascertain the effect of psychostimulant treatment on individual patients. In their systematic review, Pérez–Mañá et al. pooled the mean difference in number of negative urine screens per group as their primary outcome. Although this is an important outcome, it is statistically inappropriate to pool as a continuous outcome since the number of urine screens is a discrete value with some trials conducting as few as 10 urine tests [31]. For this reason, it is important for clinicians to identify a minimum clinically significant number of negative urine screens needed to achieve “success” in harm reduction treatment. This can allow future clinical trials to quantify the number of individuals achieving success in intervention and control groups as a dichotomous outcome and conduct a responder analysis [38]. However, this approach is also limited since there is currently no established threshold for negative urine screens needed to be “successful” in substance abuse treatment.

More than half of the trials were judged to have high risk of bias for at least one measure on the Cochrane risk of bias tool. The majority of studies did not mention allocation concealment, and incomplete outcome data (attrition) was a major source of bias. However, studies did conduct intention-to-treat analyses to compensate for the high attrition rate. Moreover, included trials had small samples (47% of trials had ≤30 participants per trial arm) and may have been underpowered to detect an effect at the individual study level. The GRADE evidence profile (Table 2) and summary of findings (Table 3) display the overall quality of evidence as assessed using the GRADE framework and can be used to appraise the certainty of findings from this review. For the outcome of abstinence, risk of bias was very serious due to lack of allocation concealment and incomplete outcome data; however, treatment retention was only affected by lack of allocation concealment. There was a relatively low heterogeneity between studies for both primary outcomes; however, existing heterogeneity was not explained by subgroup analyses given that the tests for subgroup differences were not significant. Indirectness of the population and interventions affects the quality of the evidence. Many studies had strict eligibility criteria that excluded patients with comorbid substance use disorders [13, 24–26, 28–30, 33, 35], whereas patients in clinical practice frequently present with multiple substance use disorders [39]. Furthermore, some trials were conducted in specific populations such as individuals with ADHD, incarcerated individuals, or men who have sex with men [21, 25, 26]. Psychostimulant interventions may have differential treatment effects within such specific populations diagnosed with AMD. The ORs for abstinence and treatment retention varied across studies, with little to no overlap of 95% confidence intervals of individual study point estimates. There was no evidence of publication bias for either outcomes based on visual assessment of funnel plots. Overall, the quality of evidence in the review can be considered very low based on the GRADE assessment.

**Table 2:** GRADE evidence profile

Quality assessment							No. of patients		Effect		Quality	Importance
# of studies	Study design	Risk of bias	Inconsistency	Indirectness	Imprecision	Other	Psychostimulants	Placebo	Relative (95% CI)	Absolute (95% CI)		
Abstinence from illicit amphetamines and methamphetamines (final 2 weeks of treatment) (assessed with: urinalysis)												
5	RCTs	Very serious^a,b^	Serious^c^	Serious^d^	Very serious^e^	None	70/353 (19.8%)	62/289 (21.5%)	OR 0.97 (0.65 to 1.45)	5 fewer per 1000 (from 64 fewer to 69 more)	⨁◯◯◯ Very low	Critical
Retention in treatment (end of trial) (follow-up: range 8 to 24 weeks)												
14	RCTs	Serious^b^	Serious ^c^	Very serious^d,f^	Serious^e^	None	332/626 (53.0%)	268/558 (48.0%)	OR 1.20 (0.91 to 1.58)	46 more per 1000 (from 23 fewer to 113 more)	⨁◯◯◯ Very low	Critical

*CI* confidence interval, *OR* odds ratio

^a^The majority of studies had high attrition bias (>50% dropout rate) and small sample sizes

^b^80% of studies did not mention allocation concealment, which may be a source of bias

^c^Heterogeneity was not explained by subgroup analyses, as indicated by non-significant tests for subgroup differences

^d^Studies investigating the efficacy of different psychostimulant drugs at varying doses were pooled

^e^95% confidence intervals are wide and there is a varying range of effect, with little overlap of confidence intervals from some studies

^f^Populations varied across studies with certain studies including injection drug users, incarcerated individuals, or participants with ADHD

**Table 3:** Summary of findings table

Outcomes	No. of participants (studies) Follow-up	Quality of the evidence (GRADE)	Relative effect (95% CI)	Anticipated absolute effects	
				Risk with placebo	Risk difference with psychostimulants
Abstinence from illicit amphetamines and methamphetamines (final 2 weeks of treatment) Assessed with: urinalysis	642 (5 RCTs)	⨁◯◯◯ Very low^a,b,c,d,e^	OR 0.97 (0.65 to 1.45)	215 per 1000	5 fewer per 1000 (64 fewer to 69 more)
Retention in treatment (end of trial) Follow-up: range 8 to 24 weeks	1184 (14 RCTs)	⨁◯◯◯ Very low^b,c,d,e,f^	OR 1.20 (0.91 to 1.58)	480 per 1000	46 more per 1000 (23 fewer to 113 more)

GRADE working group grades of evidence

High quality: We are very confident that the true effect lies close to that of the estimate of the effect

Moderate quality: We are moderately confident in the effect estimate: The true effect is likely to be close to the estimate of the effect, but there is a possibility that it is substantially different

Low quality: Our confidence in the effect estimate is limited: The true effect may be substantially different from the estimate of the effect

Very low quality: We have very little confidence in the effect estimate: The true effect is likely to be substantially different from the estimate of effect

The risk in the intervention group (and its 95% confidence interval) is based on the assumed risk in the comparison group and the relative effect of the intervention (and its 95% CI)

*CI* Confidence interval, *OR* Odds ratio

^a^The majority of studies had high attrition bias (>50% dropout rate) and small sample sizes

^b^80% of studies did not mention allocation concealment, which may be a source of bias

^c^Heterogeneity was not explained by subgroup analyses, as indicated by non-significant tests for subgroup differences

^d^Studies investigating the efficacy of different psychostimulant drugs at varying doses were pooled

^e^95% confidence intervals are wide and there is a varying range of effect, with little overlap of confidence intervals from some studies

^f^Populations varied across studies with certain studies including injection drug users, incarcerated individuals or participants with ADHD

### Strengths and limitations

This systematic review was conducted with an a priori design to assess the efficacy and safety of psychostimulants for AMD. We conducted a comprehensive search of psychostimulant agents and two reviewers independently conducted title and abstract screening, full text review, risk of bias assessment, and data extraction. This is the largest review conducted to date investigating psychostimulant agents for AMD including 17 RCTs; however, we were limited by the quality of evidence in individual trials. Reporting of outcomes across studies was very inconsistent and did not allow us to pool all of the available data. Furthermore, we only included published and available data from included trials rather than obtaining additional data for analyses. Published data were used in order to comment on the quality of outcome reporting in RCTs in this area. Clinician and patient important outcomes were chosen as primary and secondary outcomes for this review rather than group level outcomes reported by individual studies. Nevertheless, the small number of trials in the meta-analysis for abstinence limits the generalizability of the findings. As well, we created broad eligibility criteria to include adolescent studies, yet only one trial has been conducted that has found inversely significant results. For this reason, results of this review cannot be generalized to adolescent populations.

## Conclusions

The present systematic review found no effect of psychostimulant agents on sustained abstinence or retention in treatment among patients diagnosed with AMD. The majority of trials included participants with a diagnosis of methamphetamine dependence and were 12 weeks in treatment duration. Review findings suggest that longer duration treatment may have a favorable effect for retention in treatment. Future trials in this area should consider varying lengths of treatment (>12 weeks) to determine whether duration is associated with reduction in substance use and abstinence. The current quality of evidence is very low, and future research may have an impact on treatment outcomes. Future research should utilize standardized methods of reporting outcomes, including adverse events, such that a comprehensive summary of evidence can be produced to eventually inform clinical practice guidelines. While there are major challenges in conducting clinical trials among substance use disorder populations, identifying important outcomes in the area and consistent reporting of outcomes is integral in combining trial results to be used in making clinical recommendations. If adequate outcome reporting and high caliber methodology in future trials continues to show no efficacy of psychostimulant interventions across multiple trials, this may indicate the need to consider new treatment approaches for AMD.

## Additional files

Additional file 1: PRISMA Checklist. The PRISMA Checklist refers to the pages in which we have included the required information, as per PRISMA guidelines.
Additional file 2: Database search strategy. This document includes the comprehensive search strategy, including key terms and medical subject headings, used to search MEDLINE, EMBASE, PsycINFO, Cochrane CENTRAL, and CINAHL databases. The strategy was developed by the first author (MB) in collaboration with an expert health sciences librarian (NB).
Additional file 3: Data extraction form. The completed pilot-tested data extraction form includes the extracted details about populations, methods, interventions, comparators, outcomes and risk of bias assessment of included studies.

## Abbreviations

ADHD: Attention-deficit hyperactivity disorder
AMD: Amphetamine and methamphetamine use disorders
CI: Confidence interval
DA: Dopamine
DSM: Diagnostic and Statistical Manual of Mental Disorders
GRADE: Grading of Recommendations, Assessment, Development, and Evaluation
OR: Odds ratio
PRISMA: Preferred Reporting Items for Systematic Reviews and Meta-Analyses
RCT: Randomized controlled trial
SAE: Serious adverse event

## References

[1] American Psychiatric Association. Diagnostic and Statistical Manual of Mental Disorders (DSM-5®). 5th ed. Arlington: American Psychiatric Publishing; 2013.

[2] United Nations Office on Drugs and Crime: World Drug Report 2013. 2013.https://www.unodc.org. Accessed 06 Sept 2016.

[3] Buxton JA, Dove NA (2008). The burden and management of crystal meth use. Can Med Assoc J.

[4] Darke S, Kaye S, McKetin R, Duflou J (2008). Major physical and psychological harms of methamphetamine use. Drug Alcohol Rev.

[5] Sommers I, Baskin D, Baskin-Sommers A (2006). Methamphetamine use among young adults: health and social consequences. Addict Behav.

[6] Ciketic S, Hayatbakhsh MR, Doran CM, Najman JM, McKetin R (2012). A review of psychological and pharmacological treatment options for methamphetamine dependence. J Subst Use.

[7] Pérez-Mañá C, Castells X, Torrens M, Capellà D, Farre M. Efficacy of psychostimulant drugs for amphetamine abuse or dependence. Cochrane Database Syst Rev. 2013;9. 10.1002/14651858.CD009695.pub2 PMC11521360 23996457

[8] Lee NK, Rawson RA (2008). A systematic review of cognitive and behavioural therapies for methamphetamine dependence. Drug Alcohol Rev.

[9] Shearer J (2007). Psychosocial approaches to psychostimulant dependence: a systematic review. J Subst Abuse Treat.

[10] Grabowski J, Shearer J, Merrill J, Negus SS (2004). Agonist-like, replacement pharmacotherapy for stimulant abuse and dependence. Addict Behav.

[11] Karila L, Weinstein A, Aubin HJ, Benyamina A, Reynaud M, Batki SL (2010). Pharmacological approaches to methamphetamine dependence: a focused review. Br J Clin Pharmacol.

[12] Anderson AL, Li SH, Markova D, Holmes TH, Chiang N, Kahn R (2015). Bupropion for the treatment of methamphetamine dependence in non-daily users: a randomized, double-blind, placebo-controlled trial. Drug & Alcohol Dependence.

[13] Heinzerling KG, Swanson AN, Hall TM, Yi Y, Wu Y, Shoptaw SJ (2014). Randomized, placebo-controlled trial of bupropion in methamphetamine-dependent participants with less than daily methamphetamine use. Addiction.

[14] Moher D, Liberati A, Tetzlaff J, Altman DG (2009). Preferred reporting items for systematic reviews and meta-analyses: the PRISMA statement. Ann Intern Med.

[15] Castells X, Casas M, Pérez-Mañá C, Roncero C, Vidal X, Capellà D (2010). Efficacy of psychostimulant drugs for cocaine dependence. Cochrane Database Syst Rev.

[16] World Health Organization (1997). Anatomical therapeutical chemical classification.

[17] American Society of Hospital Pharmacists: AHFS drug information. 1990. http://www.ahfsdruginformation.com/. Accessed 06 Sept 2016.

[18] Higgins JP, Altman DG, Gøtzsche PC, Jüni P, Moher D, Oxman AD (2011). The Cochrane Collaboration’s tool for assessing risk of bias in randomised trials. BMJ.

[19] Guyatt GH, Oxman AD, Vist GE, Kunz R, Falck-Ytter Y, Alonso-Coello P (2008). GRADE: an emerging consensus on rating quality of evidence and strength of recommendations. BMJ.

[20] Guyatt G, Rennie D, Meade MO, Cook DJ (2002). Users’ guides to the medical literature: a manual for evidence-based clinical practice.

[21] Das M, Santos D, Matheson T, Santos GM, Chu P, Vittinghoff E (2010). Feasibility and acceptability of a phase II randomized pharmacologic intervention for methamphetamine dependence in high-risk men who have sex with men. Aids.

[22] Elkashef AM, Rawson RA, Anderson AL, Li SH, Holmes T, Smith EV (2008). Bupropion for the treatment of methamphetamine dependence. Neuropsychopharmacology.

[23] Galloway GP, Buscemi R, Coyle JR, Flower K, Siegrist JD, Fiske LA (2011). A randomized, placebo-controlled trial of sustained-release dextroamphetamine for treatment of methamphetamine addiction. Clinical Pharmacology & Therapeutics.

[24] Heinzerling KG, Swanson AN, Kim S, Cederblom L, Moe A, Ling W (2010). Randomized, double-blind, placebo-controlled trial of modafinil for the treatment of methamphetamine dependence. Drug Alcohol Depend.

[25] Konstenius M, Jayaram-Lindström N, Beck O, Franck J (2010). Sustained release methylphenidate for the treatment of ADHD in amphetamine abusers: a pilot study. Drug & Alcohol Dependence.

[26] Konstenius M, Jayaram-Lindström N, Guterstam J, Beck O, Philips B, Franck J (2014). Methylphenidate for attention deficit hyperactivity disorder and drug relapse in criminal offenders with substance dependence: a 24-week randomized placebo-controlled trial. Addiction.

[27] Ling W, Chang L, Hillhouse M, Ang A, Striebel J, Jenkins J (2014). Sustained-release methylphenidate in a randomized trial of treatment of methamphetamine use disorder. Addiction.

[28] Miles S, Sheridan J, Russell B, Kydd R, Wheeler A, Walters C (2013). Extended-release methylphenidate for treatment of amphetamine/methamphetamine dependence: a randomized, double-blind, placebo-controlled trial. Addiction.

[29] Longo M, Wickes W, Smout M, Harrison S, Cahill S, White JM (2010). Randomized controlled trial of dexamphetamine maintenance for the treatment of methamphetamine dependence. Addiction.

[30] Shoptaw S, Heinzerling KG, Rotheram-Fuller E, Steward T, Wang J, Swanson AN (2008). Randomized, placebo-controlled trial of bupropion for the treatment of methamphetamine dependence. Drug & Alcohol Dependence.

[31] Rezaei F, Emami M, Zahed S, Morabbi M, Farahzadi M, Akhondzadeh S (2015). Sustained-release methylphenidate in methamphetamine dependence treatment: a double-blind and placebo-controlled trial. Daru.

[32] Tiihonen J, Kuoppasalmi K, Föhr J, Tuomola P, Kuikanmäki O, Vorma H (2007). A comparison of aripiprazole, methylphenidate, and placebo for amphetamine dependence. Am J Psychiatry.

[33] Heinzerling KG, Gadzhyan J, van Oudheusden H, Rodriguez F, McCracken J, Shoptaw S (2013). Pilot randomized trial of bupropion for adolescent methamphetamine abuse/dependence. J Adolesc Health.

[34] Shearer J, Darke S, Rodgers C, Slade T, van Beek I, Lewis J (2009). A double-blind, placebo-controlled trial of modafinil (200 mg/day) for methamphetamine dependence. Addiction.

[35] Anderson AL, Li SH, Biswas K, McSherry F, Holmes T, Iturriaga E (2012). Modafinil for the treatment of methamphetamine dependence. Drug Alcohol Depend.

[36] Hubbard RL, Craddock SG, Anderson J (2003). Overview of 5-year followup outcomes in the drug abuse treatment outcome studies (DATOS). J Subst Abuse Treat.

[37] Perez-Mana C, Castells X, Vidal X, Casas M, Capella D (2011). Efficacy of indirect dopamine agonists for psychostimulant dependence: a systematic review and meta-analysis of randomized controlled trials. J Subst Abuse Treat.

[38] Winchell C, Rappaport BA, Roca R, Rosebraugh CJ (2012). Reanalysis of methamphetamine dependence treatment trial. CNS Neurosci Ther.

[39] Dennis BB, Roshanov PS, Naji L, Bawor M, Paul J, Plater C, Pare G, Worster A, Varenbut M, Daiter J (2015). Opioid substitution and antagonist therapy trials exclude the common addiction patient: a systematic review and analysis of eligibility criteria. Trials.

