# Supplementary material for: Efficacy and safety of psychostimulants for amphetamine and methamphetamine use disorders: a systematic review and meta-analysis
Source: Syst Rev. 2016 Nov 14;5:189. doi: 10.1186/s13643-016-0370-x (PMC5109734; doi:10.1186/s13643-016-0370-x)
Supplement: Additional file 2: — Database search strategy. This document includes the comprehensive search strategy, including key terms and medical subject headings, used to search MEDLINE, EMBASE, PsycINFO, Cochrane CENTRAL, and CINAHL databases. The strategy was developed by the first author (MB) in collaboration with an expert health sciences librarian (NB). [file 13643_2016_370_MOESM2_ESM.docx]

**SEARCH STRATEGY**

**OVID MEDLINE In-Process & Other Non-Indexed Citations**

1. exp Amphetamine-Related Disorders/
2. ((amphetamine OR amfetamine OR methamphetamine) ADJ (dependen* OR addict* OR abuse OR misuse)).ti,ab.
3. exp Amphetamine/
4. exp Methamphetamine/
5. exp Central Nervous System Stimulants/
6. (amphetamine OR amfetamine OR acefylline piperazine OR adrafinil OR amfebutamone OR amfepramone OR aminorex OR aminophylline OR bamifylline OR benzphetamine OR bufylline OR bupropion OR caffeine OR cathine OR cathinone OR choline theophyllinate OR citicoline OR clobenzorex OR dexamphetamine OR dexanfetamine OR dexmethylphenidate OR diethylpropion OR diprophylline OR doxofylline OR dyphylline OR ephedrine OR etamiphylline OR ethylamphetamine OR fencamfamine OR fenetylline OR fenozolone OR lisdexanfetamine OR mazindol OR mefenorex OR mesocarb* OR methamphetamine OR methylenedioxymethamphetamine* OR methylphenidate OR modafinil OR nicotine OR norpseudoephedrine OR pemoline OR phentermine OR pipradrol OR prolintane OR propentofylline OR proxyphylline OR selegiline OR sydnocarb OR theobromine OR theophylline).mp.
7. 1 OR 2
8. 3 OR 4 OR 5 OR 6
9. 7 AND 8
10. (randomized controlled trial OR clinical trial).pt.
11. random*.ti,ab.
12. 10 OR 11
13. 9 AND 12
14. limit 13 to humans

**EMBASE**

1. exp Amphetamine-Related Disorders/
2. ((amphetamine OR amfetamine OR methamphetamine) ADJ (dependen* OR addict* OR abuse OR misuse)).ti,ab.
3. exp Amphetamine/
4. exp Methamphetamine/
5. exp Central Nervous System Stimulants/
6. (amphetamine OR amfetamine OR acefylline piperazine OR adrafinil OR amfebutamone OR amfepramone OR aminorex OR aminophylline OR bamifylline OR benzphetamine OR bufylline OR bupropion OR caffeine OR cathine OR cathinone OR choline theophyllinate OR citicoline OR clobenzorex OR dexamphetamine OR dexanfetamine OR dexmethylphenidate OR diethylpropion OR diprophylline OR doxofylline OR dyphylline OR ephedrine OR etamiphylline OR ethylamphetamine OR fencamfamine OR fenetylline OR fenozolone OR lisdexanfetamine OR mazindol OR mefenorex OR mesocarb* OR methamphetamine OR methylenedioxymethamphetamine* OR methylphenidate OR modafinil OR nicotine OR norpseudoephedrine OR pemoline OR phentermine OR pipradrol OR prolintane OR propentofylline OR proxyphylline OR selegiline OR sydnocarb OR theobromine OR theophylline).mp.
7. 1 OR 2
8. 3 OR 4 OR 5 OR 6
9. 7 AND 8
10. randomized controlled trial/
11. clinical trial/
12. 10 OR 11
13. 9 AND 12
14. limit 13 to humans

**PSYCInfo**

1. ((amphetamine OR amfetamine OR methamphetamine) ADJ (dependen* OR addict* OR abuse OR misuse)).ti,ab.
2. exp Amphetamine/
3. exp Methamphetamine/
4. exp Central Nervous System Stimulants/
5. (amphetamine OR amfetamine OR acefylline piperazine OR adrafinil OR amfebutamone OR amfepramone OR aminorex OR aminophylline OR bamifylline OR benzphetamine OR bufylline OR bupropion OR caffeine OR cathine OR cathinone OR choline theophyllinate OR citicoline OR clobenzorex OR dexamphetamine OR dexanfetamine OR dexmethylphenidate OR diethylpropion OR diprophylline OR doxofylline OR dyphylline OR ephedrine OR etamiphylline OR ethylamphetamine OR fencamfamine OR fenetylline OR fenozolone OR lisdexanfetamine OR mazindol OR mefenorex OR mesocarb* OR methamphetamine OR methylenedioxymethamphetamine* OR methylphenidate OR modafinil OR nicotine OR norpseudoephedrine OR pemoline OR phentermine OR pipradrol OR prolintane OR propentofylline OR proxyphylline OR selegiline OR sydnocarb OR theobromine OR theophylline).mp.
6. 2 OR 3 OR 4 OR 5
7. 1 AND 6
8. random*.ti,ab.
9. 7 AND 8
10. limit 9 to human

**Cochrane CENTRAL Trials Registry**

#1 MeSH descriptor: [Amphetamine-Related Disorders] explode all trees

#2 ((amphetamine or amfetamine or methamphetamine) next (dependen* or addict* or abuse or misuse or disorder))

#3 MeSH descriptor: [Central Nervous System Stimulants] explode all trees

#4 MeSH descriptor: [Amphetamine] explode all trees

#5 MeSH descriptor: [Methamphetamine] explode all trees

#6 amphetamine* or amfetamine* or acefylline piperazine or adrafinil or amfebutamone or amfepramone or aminorex or aminophylline or bamifylline or benzphetamine or bufylline or bupropion or caffeine or cathine or cathinone or choline theophyllinate or citicoline or clobenzorex or dexamphetamine or dexanfetamine or dexmethylphenidate or diethylpropion or diprophylline or doxofylline or dyphylline or ephedrine or etamiphylline or ethylamphetamine or fencamfamine or fenetylline or fenozolone or lisdexanfetamine or mazindol or mefenorex or mesocarb* or methamphetamine or methylenedioxymethamphetamine or methylphenidate or modafinil or nicotine or norpseudoephedrine or pemoline or phentermine or pipradrol or prolintane or propentofylline or proxyphylline or selegiline or sydnocarb or theobromine or theophylline

#7 central nervous system stimulant* or psychostimulant* or cns stimulant* in keywords

#8 #3 or #4 or #5 or #6 or #7

#9 #1 or #2

#10 #8 and #9 in Trials

**CINAHL**

| S19 | S9 AND S17 AND S18 |
| --- | --- |
| S18 | S10 OR S11 OR S12 OR S13 OR S14 |
| S17 | S15 AND S16 |
| S16 | TI dependen* OR TI addict* OR TI abuse OR TI misuse OR TI disorder OR AB dependen* OR AB addict* OR AB abuse OR AB misuse OR AB disorder |
| S15 | TI amphetamine OR AB amphetamine OR TI amfetamine OR AB amfetamine OR TI methamphetamine OR AB methamphetamine |
| S14 | TI trial OR AB trial |
| S13 | TI randomly OR AB randomly |
| S12 | TI ( randomised or randomized ) OR AB ( randomised or randomized ) |
| S11 | TI placebo OR AB placebo |
| S10 | (MH "Randomized Controlled Trials") OR (MH "Clinical Trials+") |
| S9 | S1 OR S2 OR S3 OR S4 OR S5 OR S6 OR S7 OR S8 |
| S8 | "psychostimulant*" |
| S7 | "central nervous system stimulant*" |
| S6 | "cns stimulant*" |
| S5 | AB amphetamine* or amfetamine* or acefylline piperazine or adrafinil or amfebutamone or amfepramone or aminorex or aminophylline or bamifylline or benzphetamine or bufylline or bupropion or caffeine or cathine or cathinone or choline theophyllinate or clobenzorex or dexamphetamine or dexanfetamine or dexmethylphenidate or diethylpropion or diprophylline or doxofylline or dyphylline or ephedrine or etamiphylline or ethylamphetamine or fencamfamine or fenetylline or fenozolone or lisdexanfetamine or mazindol or mefenorex or mesocarb* or methamphetamine or methylenedioxymethamphetamine* or methylphenidate or modafinil or nicotine or norpseudoephedrine or pemoline or phentermine or pipradrol or prolintane or propentofylline or proxyphylline or selegiline or sydnocarb or theobromine or theophylline |
| S4 | "psychostimulant*" |
| S3 | (MH "Central Nervous System Stimulants+") OR (MH "Central Nervous System Agents+") |
| S2 | (MH "Methamphetamine+") |
| S1 | (MH "Amphetamine+") OR (MH "Amphetamines+") |
